# Emerging Roles for G-protein Coupled Receptors in Development and Activation of Macrophages

**DOI:** 10.3389/fimmu.2019.02031

**Published:** 2019-08-27

**Authors:** Xinming Wang, Abishek Iyer, A. Bruce Lyons, Heinrich Körner, Wei Wei

**Affiliations:** ^1^Key Laboratory of Anti-inflammatory and Immune Medicine, Anhui Collaborative Innovation Center of Anti-inflammatory and Immune Medicine, Institute of Clinical Pharmacology, Ministry of Education, Anhui Medical University, Hefei, China; ^2^Department of Pharmacy, First Affiliated Hospital of Anhui Medical University, Hefei, China; ^3^Institute for Molecular Bioscience, University of Queensland, Brisbane, QLD, Australia; ^4^School of Medicine, University of Tasmania, Hobart, TAS, Australia; ^5^Menzies Institute for Medical Research, University of Tasmania, Hobart, TAS, Australia

**Keywords:** G-protein coupled receptors, macrophages, differentiation, polarization, innate immunity

## Abstract

Macrophages have emerged as a key component of the innate immune system that emigrates to peripheral tissues during gestation and in the adult organism. Their complex pathway to maturity, their unique plasticity and their various roles as effector and regulatory cells during an immune response have been the focus of intense research. A class of surface molecules, the G-Protein coupled receptors (GPCRs) play important roles in many immune processes. They have drawn attention in regard to these functions and the potential for therapeutic targets that can modulate the response of immune cells in pathologies such as diabetes, atherosclerosis, and chronic inflammatory diseases. Of the more than 800 GPCRs identified, ~100 are currently targeted with drugs which have had their activity investigated *in vivo*. Macrophages express a number of GPCRs which have central roles during cell differentiation and in the regulation of their functions. While some macrophage GPCRs such as chemokine receptors have been studied in great detail, the roles of other receptors of this large family are still not well understood. This review summarizes new insights into macrophage biology, differences of human, and mouse macrophages and gives details of some of the GPCRs expressed by this cell type.

## Introduction

Inflammation is a highly complex protective response of the mammalian immune system that is initiated at sites of infection or injury. The main goal of this process is to remove the danger stimulus and damaged cells, resolve inflammation, and support a repair of the damaged tissue. However, if this process that is normally targeted at an infectious stimuli or injured tissue remains unresolved, innate immune activation can be prolonged and become misdirected at healthy cells leading to chronic inflammation and disease (1). Central to the inflammatory response and its regulation is the cellular innate immune response that in its early stages encompasses an initial neutrophil influx into the tissue followed by an immigration of monocytes and cells of the adaptive immune system. Inflammatory monocytes differentiate and form a major cellular component, the macrophages. Macrophages respond strongly and early to antigenic challenges in the tissue and the local cytokine environment. Their central role in the regulation of an effective immune response is being increasingly appreciated and their dysregulation in this early phase can lead to various human diseases that are associated with chronic inflammation (1). Thus, controlling macrophage-driven immune responses is an important preventative and therapeutic goal in many chronic inflammatory pathologies.

Macrophages have central roles in danger detection, inflammation, and host defense (2). These innate immune cells sense danger signals in their environment, inhibit or kill pathogens, engulf apoptotic, and necrotic cells and present antigens to adaptive immune cells (PMID:27313577). They have evolved to detect various pathogen-associated molecular patterns (PAMPs) and damage-associated molecular patterns (DAMPs) in their environment and communicate with other keys cells of the innate and adaptive immune system via an array of cell surface receptors (3). For example, Toll-like receptors (TLRs) are the most extensively studied pattern recognition receptors that play crucial roles in regulating macrophage biology and innate immunity (4). TLRs sense a variety of extracellular and intracellular PAMPs and DAMPs and activation of TLR-mediated cell signaling constitutes one of the earliest response to danger detection in macrophages (4). Similar to TLRs, a separate family of even more diverse sentinel cell-surface receptors, the G-protein-Coupled Receptors (GPCRs) (5), allows the cell to perceive and sense pathogen and damage-associated danger signals integrating environmental signals with cellular signaling networks. These GPCRs act as gate-keepers in general and help in particular to shape immune responses of macrophages toward extracellular pathogens as well injury related danger molecules (6, 7). For example, the mammalian immune system has evolved GPCRs as part of the complement cascade that plays a critical, protective role in dealing with microbial intruders (8). However, many mammalian pathogens have developed innovate ways to escape being targeted by the complement cascade through a range of different mechanisms including the manipulation of macrophage GPCR signaling (8, 9). Moreover, a large number of studies have demonstrated clearly that mammalian GPCRs feature prominently in the regulation of other aspects of macrophage biology including macrophage development, differentiation and activation. Understanding the key roles played by macrophage GPCRs will help in designing effective therapies for a range of chronic human inflammatory pathologies.

The human genome encodes more than 800 GPCRs that bind to a large variety of endogenous ligands which control many physiological processes and are major drug targets. Nearly 35 percent of all clinically used drugs target GPCRs (10). Furthermore, many new GPCR-targeting drugs are currently undergoing clinical trials in human patients or are in the last stages of approval (10). With the recent emergence of three dimensional crystal and cyro-electron microscopy structures for several GPCRs (11), this family of cell surface receptors promises many new opportunities for the development of new, more effective therapeutic agents. Therefore, this review aims to document current literature and present examples of how GPCRs may play major roles in regulating various macrophage functions in development, differentiation and activation. We will also discuss examples of how targeting GPCR in macrophages could potentially be harnessed for treating a wide variety of both acute and chronic human inflammatory diseases.

## New Insights in Macrophage Biology

Macrophages together with monocytes comprise the mononuclear phagocyte system (MPS) (12) [historically named the reticuloendothelial system (13)] and are members of the hematopoietic linage. Initially, it was assumed that tissue macrophages originated from committed precursor cells that differentiated in the bone marrow, entered the periphery and replenished the various tissue specific populations by immigration of circulating peripheral monocytic cells (12). A specialized antigen presenting cell type termed dendritic cells was added later to the MPS (14, 15). The increasing complexity of the MPS and the interconnection of its various components made it soon clear that the initial simplistic models required updating. The historical development of the field of macrophage research including the underlying experimental evidence and the various iterations of the terminology has been detailed in a recent review (16).

New technologies such as flow cytometry, confocal microscopy, the genetic analysis of single cells, and fate mapping by means of introducing dyes into the germ-line of specific cell types have allowed major advances in our understanding of MPS ontology. Our current understanding is that during embryonic development, most tissues are seeded by precursor cells in two waves that originate from yolk sac and fetal liver, respectively. In the adult organism these tissue resident macrophages propagate locally and largely remain in homeostasis independently of a replenishment (17, 18). The CNS-resident microglia (19) and skin-resident Langerhan's cells (20) are derived directly from yolk sac progenitors. These cells are among the earliest immigrants in embryonic tissues (18) and while microglial cells show hardly any turn over in adult tissues the resident Langerhan's dendritic cells of the skin are replaced successively during adult life. Other tissue-resident macrophages such as Kupffer cells (liver), alveolar macrophages (lung), and skin macrophages are derived from fetal liver hematopoietic cells and show a combination of proliferation and very limited turnover under homeostasis (17). An exception is intestinal macrophages which are originally derived from fetal liver but are replaced soon after birth by bone marrow-derived monocytes (21, 22). This process is microbiota driven and the influx is maintained throughout adult life (23).

Committed Monocyte-DC precursors (MDP) are constantly developing in the bone marrow (24) and replenish two mutually exclusive lines of differentiation that lead to either monocytic (25) or dendritic cell precursors (26, 27) both of which have access to the periphery. Two chemokine receptors, CCR2, and CX3CR1 are intimately connected with the development of macrophages. Inflammatory as well as steady-state monocytes leave the bone marrow as CD115^+^ CX_3_CR1^int^ Ly6C^high^ monocytes, guided by the chemokine receptor CCR2 (28). Depending on the cytokine environment and the nature and kinetics of the acute inflammation, these cells can differentiate into iNOS^+^ phagocytes as shown originally in the Listeria monocytogenes model (29). Alternatively, these cells can differentiate into inflammatory, monocytic DCs (moDCs) (30). The regulation of these pathways has been attributed to an intrinsic heterogeneity of the PU.1 expression of the initial precursor cell population (31).

While steady state conditions favors a tissue-resident, self-replicating macrophage compartment with minimal input from the periphery, inflammatory conditions quickly result in a strong influx of peripheral bone marrow (BM)-derived monocytes which enter the tissue as inflammatory effector cells. Depending on nature and kinetics of the specific inflammatory response and the given cytokine environment, monocytes can rapidly develop into inflammatory effector M1 macrophages producing, in mouse and humans, molecules such as TNF and iNOS (32). Alternatively, contradicting the notion that a differentiation to tissue macrophages is a default pathway, they can recirculate back to the lymph node (LN) with minimal differentiation (33). While under certain circumstances they can take on the appearance of the replaced tissue macrophages in some tissues (e.g., liver) (34) these cells commonly fail to replace the original tissue resident macrophages. After the elimination of the inflammatory cues as the acute immune response retracts, the majority of these cells die (35). A small proportion of monocyte-derived macrophages which survive for reasons unknown display remarkable plasticity and can differentiate directly to M2 macrophages (36). These macrophages express a different set of effector molecules and have been described to be involved in a different set of activities, such as tissue repair. While the concept of a M1/M2 polarization was initially formulated to explain differences in the way macrophages generated *in vitro* responded to different stimuli it has now been widely accepted that under physiological conditions *in vivo*, similar polarization occurs (32, 37).

The heterogeneity of the local macrophage compartment and the consequential complexity and integration of the various cell populations has been highlighted in a detailed investigation of the myeloid cells in the skin (38). Using two marker molecules, CD64 and CCR2, five populations of macrophages and dendritic cells with an array of different functions from phagocytosis to antigen presentation have been identified (38). This points to a major limitation of many current studies based on an analysis of tissue samples. Only the analysis of sorted cell populations or, preferably, single cell analysis of a representative number of cells can resolve this heterogeneity. A further caveat with regard to an analysis of monocytes of macrophage populations in periphery or tissue is the plasticity and responsiveness of the myeloid cells. Every analysis, especially during an inflammatory response, has to be seen as a snapshot of a transient situation (39). Within days, or even hours, proportions populations and differentiation status may have changed dramatically. A final reservation with regard to the analysis of local macrophage populations is the ongoing lack of an agreed set of marker molecules that allow to sufficiently describe any given myeloid population in steady state and inflammation (40).

## Human and Mouse Macrophages: How Similar Are They?

The question of the degree of similarity between the inflammatory response in general and cells of the MPS in particular in either mouse or human is only partly answered and many aspects have not yet been elucidated (41). While macrophage research in rodents is frequently based on either thioglycolate-elicited peritoneal or bone marrow-derived macrophages, in humans blood-derived monocytes are easily harvested and therefore usually used for investigations after cytokine-driven maturation (42). Nevertheless, there have been some exceptions to this relatively common approach and some progress in the alignment of these two systems has been made. In a genetic approach, mouse and human blood monocytes were isolated and cultured identically. This resulted in a true comparison of conserved and non-conserved marker molecules and showed, for example, that CD64 was elevated as a surface marker on the comparable Ly6C^+^ mouse and CD16^−^ human monocyte populations. However, in contrast to being weakly positive on mouse Ly6C^low^ monocytes, CD64 had disappeared from human CD16^+^ monocytes. Other molecules such as MHC class II were clearly differentially regulated in both species with human monocytes uniformly positive for this marker while mouse monocytes were almost entirely negative without an activating stimulus (43). While this genetic comparison allows precise delineations of gene expression, this analysis neglects the functional aspect. Consequently, other attempts to align various monocyte populations have relied on comparative functional studies. Isolation and functional analysis of CD14^dim^ monocytes showed that they constituted a homolog of the “patrolling” murine Ly6C^low^ monocyte subset (44). Finally, an interesting approach to elucidate the role of certain monocyte populations in humans is the analysis of human inherited diseases such as autosomal dominant and sporadic monocytopenia which results in an absence of myeloid cells amongst others, in the periphery (45). A dissection of the myeloid cell populations present or absent gave striking insight in the ontology of the human MPS (46). Despite an absence of peripheral monocytes, dermal macrophages and Langerhan's cells were not impacted by this disorder, as predicted by mouse experiments.

## Structure and Classification of GPCRs

GPCRs are ligand-coupled proteins that are found in the transmembrane region of the cell surface and function primarily as transducers of extracellular stimuli into intracellular signals that elicit cellular responses. In humans, over 800 GPCRs have been identified in the genome (>3% of human genome) and are associated with various ligands such as hormones, neurotransmitters, growth factors, chemokines, ions, odorant molecules, and even light photons (5). These ligands are known to bind to only around 200–300 GPCRs, while the remaining GPCRs are considered orphan receptors as no ligand or function has been identified so far. Many of the remaining GPCRs are thought to be sensory in function (47). GPCRs are central to various physiological functions and dysregulated expression and signaling of GPCRs has been associated with various chronic inflammatory diseases such as rheumatoid arthritis (48) or diseases with certain inflammatory aspects such as cancer (49, 50). This makes GPCRs important targets for development of new therapeutics and is reflected by over 35 percent of all clinically marketed drugs designed and developed to modulate their function. However, registered drugs have only been produced for about 30–40 well-characterized GPCRs and therefore, there are many opportunities to validate and discover new drug targets and therapeutics from the remaining GPCRs (10).

GPCRs have a common structure of seven transmembrane α-helical segments (H1–H7) joined with three intracellular (I1, I2, and I3) and three extracellular (E1, E2, and E3) loops, an extracellular N-, and an intracellular C-terminus. The crystallization and structural analysis of GPCRs including rhodopsin, β1 adrenergic, β2 adrenergic, A2A adenosine, glucagon, glucagon-like peptide, corticotropin-releasing factor, metabotropic glutamate, and smoothen receptors has provided new insights into the structure, mechanism, and regulation of this class of receptors (11). Based on earlier studies (51, 52) the International Union of Basic and Clinical Pharmacology Committee on Receptor Nomenclature and Drug Classification has categorized GPCRs into 5 different families, which are class A (rhodopsin-like receptors), class B (secretin-like receptors), class C (glutamate- like receptors), frizzled, and other 7TM protiens (47). Class A also known as rhodopsin-like receptors is the largest and most diverse group amongst the five families and consists of receptors for light, olfactory, biogenic amines, chemokines, prostanoids, adrenaline, and many others. Class B includes receptors for the parathyroid hormone, calcitonin and the diverse family of gastrointestinal hormones such as glucagon and secretin. Class C GPCRs consists of the GABAB receptor, calcium-sensing receptor and the family of metabotropic glutamate receptors. Class C is relatively small and members generally contain a large extracellular amino terminus thought to be important for ligand capture (47). Despite the general sequence and structural similarities of members within each family (over 25%), individual GPCRs have prominent differences in their extracellular and intracellular loops and these regions are important for ligand binding and interaction with downstream mediators (53). These differences allow individual GPCRs to exhibit unique signaling properties due to different receptor couplings to different G proteins, resultant difference intracellular pathway signaling, different G protein independent pathway activation, as well as complex regulatory processes such as receptor desensitization, internalization, endocytosis, and re-sensitization (53). An updated list of human GPCRs and their ligands has been provided by a committee of the International Union of Pharmacology (47) and a frequently updated webpage can be interrogated for the newest relevant information (https://gpcrdb.org/).

## Overview of GPCRs-mediated Cell Signaling

The molecular structure, function and signaling of GPCRs have been covered in detail before and will only be discussed briefly (5, 11, 54, 55). Activation signals leading to a conformational change in GPCRs causes the disassociation of a group of proteins located at the cytoplasmic face of the plasma membrane that receives, interprets and directs signals to diverse sets of downstream target proteins. GPCRs are coupled to four families of heterotrimeric α*βγ* guanine nucleotide-binding proteins (G proteins) and ligand-induced dissociation of the components triggers activation or inhibition of a range of different intracellular effector systems (e.g., adenylate cyclase, phospholipase C, phosphodiesterases, Ca^2+^ mobilization, others) that regulate cell function (5, 11, 54, 55). There are currently four known distinct subfamilies of heterotrimeric Gα proteins (Gi/o, Gs, Gq/11, and G12/13) (56) and activation and disassociation of these proteins are the first step in the GPCR signaling axis immediately downstream of activated receptors (5, 11, 54, 55). Activation of Gαi proteins inhibits adenylyl cyclase, while Gαs activates adenylyl cyclase mediated cell signaling pathways. Gαq activates phospholipase Cβ (PLCβ)-mediated pathways and Gα12/13 that stimulate Rho guanine-nucleotide exchange factors (5, 11, 54, 55). Activation of GPCRs by agonists mediates an elusive conformational change to the receptor that promotes the exchange of GDP for GTP in the α-subunit of the associated G protein. This leads to the activation of the G protein and the dissociation of its βγ dimer and α-subunit. Gβγ subunits also regulate crucial signaling pathways and cellular functions including effectors such as phosphatidylinositol-4,5-bisphosphate 3-kinase (PI3K), and downstream AKT signaling (11, 54, 55). G-protein signaling is terminated via receptor desensitization and internalization. Desensitization of GPCRs is initiated by phosphorylation of intracellular residues by GPCRs kinases that induce recruitment and subsequent interactions with other cytosolic β-arrestin proteins promoting internalization (57–60). In some cases, internalized GPCRs may promote a subsequent round of β-arrestin-mediated MAPK-ERK signaling (57–60). Apart from internalization and signaling, β-arrestin activation also regulates GPCRs trafficking and receptor recycling (57–60). This diverse and interconnected cell signaling pathways involved in GPCR-regulated biological responses definitely make the discovery of new therapeutics a challenging and ever-growing field. Nonetheless, a holistic understanding of GPCR cell signaling pathways and the discovery of antagonists for some of the GPCRs that have specific roles in driving inflammatory responses in macrophages and other immune cells will hopefully produce newer, more effective therapeutic agents for many chronic inflammatory diseases.

## Macrophages and GPCRs in Murine and Human Biology

The putative roles of GPCRs in differentiating and mature macrophages, and in steady state and inflammation have been summarized previously (61). Since the publication of the above study, the expression of GPCRs has been determined in human primary monocytes and blood-derived macrophages in the context of M1 vs. M2 polarization (62) or using GM-CSF or CSF-1 as opposing cytokine stimuli (63). A comprehensive experimental overview has addressed both aspects (7). Most profiled GPCRs were downregulated after differentiation from monocyte to macrophage but this study highlighted a set of seven GPCRs with upregulated expression after this first step and 15 GPCRs that were differently expressed after exposure to different cytokine regimes without any further activation. Finally, LPS activation identified further sets of GPCRs that responded differently to this stimulus (7). Since this is the most comprehensive study of regulation of GPCRs in macrophage differentiation and inflammation we used it to guide us to document emerging roles for GPCRs in regulating macrophage biology (Table 1).

**Table 1:** GPCRs expressed on macrophages.

**Full name**	**Receptor abbreviation**	**Class**	**Function (macrophage)**	**References**
Sphingosine-1-phosphate receptor	S1PR1 (EDG1), S1PR2 (EDG5) and S1PR4 (EDG6)	Class A	Macrophage activation (promotes M2) and immune regulation, migration	(64–66)
Leucine-rich repeat-containing G-protein coupled receptor	LGR1-4 GPCRs1-4	Class A	Macrophage activation (promotes M2) and immune regulation	(67)
Chemokine receptors	CR, CCR, CXCR, CX_2_CR, CX_3_CR	Class A	Cell migration during macrophage development and inflammation, activation	(17, 29, 68)
Purinergic receptors	P2Y purinoceptor family; Adenosine or P1 receptor family (AR_A1_-AR_A3_)	Class A	Pro- and anti-inflammatory macrophage activation	(69)
Complement protein 3a receptor	C3aR	Class A	Innate immunity, metabolic regulation, immune regulation	(70)
Complement protein 5a receptor	C5aR	Class A	Macrophage activation (M1) and immune regulation, migration.	(71–73)
Protease-activated receptors	PAR1, PAR2, PAR3, PAR4	Class A	Macrophage activation (M1) and immune regulation	(74–76)

### Sphingosine-1-Phosphate Receptor Family Members S1PR1 (EDG1), S1PR2, and S1PR4 (EDG6)

Members of the sphingosine-1-phosphate (S1P) receptor family have been identified to have major roles in the immune response. In a landmark study of the function of S1PR1 using a gene deficient mouse model and treatment with the immunosuppressive drug FTY720 (a S1PR1 agonist which is also known as fingolimod), it was demonstrated that S1PR1 was involved in the regulation of T cell egress from the thymus and lymphocyte egress from peripheral lymph nodes (77). The lymphocyte retention in lymph nodes caused by FTY-720 led to lymphopenia in the periphery. Further analysis showed that the lympopena was only one effect of FTY-720. Treatment of mice with the drug caused an accumulation of S1PR-positive CD68+ macrophages in the subcapsular sinus of mesenteric lymph nodes which could be confirmed using flow cytometry while the proportion of peripheral monocytes remained unchanged (78). S1PR1 and a second sphingosine-1-phosphate receptor S1PR4 and are highly regulated during macrophage differentiation. Both receptors showed an opposing regulation in CSF-1- vs. GM-CSF-differentiated macrophages (7). FTY720 is phosphorylated *in vivo* by sphingosine kinase and mimics sphingosine- 1-phosphate (S1P) but is causing receptor internalization and receptor downregulation (79). Thus, it can act as an agonist for four of the five members of the S1P family of GPCRs. Treatment of human microglia, monocyte-derived DCs and macrophages with FTY720 affects the subpopulations differently. It blocks a CD40L-induced IL-12p70 production by DCs and macrophages but not microglia and increases IL-10 expression exclusively in microglia. Therefore, it shifts the outcome of macrophage polarization to an anti-inflammatory type (64). Interestingly, an addition of the S1PR1-3 antagonist VPC44116 also blocked the expression of proinflammatory cytokines confirming the important role S1PR1 has in anti-inflammatory macrophage polarization (65). In EAE, the severity of clinical disease was correlated with S1PR1 overexpression on myeloid cells (80) while a downregulation of S1PR1 on astrocytes and thus presumably non-immunological mechanisms of FTY720 were identified as contributing to a positive therapeutic effect (81). Under the trade name Gilenya (Novartis) FTY720 has been approved in 2011 for treatment of relapsing/remitting MS. While S1PR2-deficiency does not have an impact on expression of proinflammatory cytokines (65) it also causes a significant accumulation of CD11b^+^ F4/80^+^ inflammatory macrophages during experimental peritonitis indicating that S1P acts as a negative regulator of macrophage infiltration (66). Blocking of S1PR2 by genetic or pharmaceutical means in an EAE model reduced the number of cells positive for the microglia/macrophage marker ionized calcium binding adaptor molecule 1 (*Iba1*), attenuated demyelination, and improved clinical outcome (82). This result underlines the importance of this second receptor *in vivo*.

### Leucine-Rich Repeat-Containing G-protein Coupled Receptor 4 (LGR4)

LGR4 is a Class A receptor (10) which together with the related receptors LGR1−3 responds to proteins of the R-spondin family, a group of 4 secreted ligands that regulate the canonical Wnt/beta-catenin signaling pathway (83). The expression pattern of LGR4 in human tissues mapped by IHC analysis supports a role in the development and maintenance of skin, kidney and the reproductive systems (84). In comparison to monocytes LGR4 was upregulated in both GM-CSF and CSF-1 stimulated macrophages (7). A recent study has highlighted the role of LGR4 in M2 polarization. The genetic targeting of this receptor decreased the number of tumor-associated M2 macrophages (TAM) together with reduced tumor growth in mouse tumor models (67). This switch in macrophage polarization could result in an exciting new therapeutic target that supports checkpoint therapies in lung cancer such as anti-PD-1 therapy.

### Chemokine Receptors

Chemokin receptors belong to the class A family of GPCRs (10). A cluster of five chemokine receptors (CCR2, CCR5, CCR7, CX3CR1, and FPR1) is strongly expressed on monocytes (62). While most of these GPCRs are crucial for the guided movement of cells of the immune system and are therefore of pharmaceutical interest (68) especially two chemokine receptors, CCR2 and CX3CR1 which contribute to monocyte emigration from the bone marrow and support monocytes patrolling vessels, respectively, is worth mentioning in the context of this review. In particular the pro-inflammatory axis between CCL2 and its cognate receptor CCR2 has been dissected in numerous models and while in models of atherosclerosis and MS promising results have been obtained other models have shown that there numerous caveats and more work needs to be done to fully understand the role of this receptor in inflammation. It could be demonstrated that monocyte accumulation in the atherosclerotic plaque is driven by different chemokine receptors such as CCR2 and CX3CR1 (85). Absence of CCR2 in a mouse model of severe atherosclerosis reduced the formation of lesions (86). Furthermore, a strong experimental case for an important role of CCR2 (87) and CX3CR1 (88) was made in a model of MS EAE using reporter mice that allowed an identification of microglia and inflammatory monocytes and their functions in the CNS (89). These reporter mice showed that microglia expressed CX3CR1 from the embryonic stage to adulthood and that the majority of inflammatory monocytes were CCR2^+^. In both models an intervention in the CCL2-CCR2 axis seems to be promising as a therapy and drugs are currently undergoing clinical trials to determine their potential. However, in a mouse model of Alzheimer CCR2-deficiency accelerate Alzheimer-like symptoms in El Khoury et al. (90) and in a model of age-related macular degeneration CCR2 and CCL2-deficient mice developed increased symptoms of the disease (91).

### Purinergic Receptors

Purinergic receptors consist of two families in the class A subgroup of GPCRs that are nearly ubiquitously expressed at low level and are activated either by Adenosine (P1 or Adenosine receptors) or nucleotides (ATP, ADP, UTP, and UDP; P2Y receptors). The family of P1 or adenosine receptors consists of 4 receptors (A1, A2A, A2B, and A3) encoded by separate genes (92). Extracellular Adenosine is present in acute and chronic inflammation and is used as a danger signal. Adenosine Receptor A2A and A3 are especially interesting due to their ubiquitous expression after cellular activation and their role in macrophage polarization (69). Currently intensive research for antagonists is ongoing with a special focus on cancer therapy (93, 94). P2Y receptors are a subfamily of eight purinergic G-protein-coupled receptors for adenosine and uridine nucleotides that belongs to the Class A family (10). Receptors of this family are activated by extracellular ATP and modify the inflammatory activation of macrophages (69). P2Y purinoceptor 8 (P2RY8) was highly enriched in macrophages after CSF-1 activation (7). A second GPCRs of this family, P2ry12, has been identified as a signature gene of homeostatic microglia (95). While other receptors of this group have been targeted for drug development (1, 4, 11, 13, and 14), these receptors have not yet been explored as drug targets (10).

### Complement Protein 3a Receptor (C3aR)

The “Complement system” is an ancient and conserved protein network of the immune system that is activated through proteolytic cascades by serine proteases in a highly coordinated and controlled fashion (70). Among complement proteins, complement peptide C3a acts via an inflammatory GPCR to trigger immune cell chemotaxis and/or inflammatory responses (70). The complement peptide C3a is one of the key chemoattractants and it specifically mediates chemotactic effects by binding to the C3aR expressed on various immune and non-immune cells such as monocytes, macrophages, mast cells, eosinophils, stem cells, and many others. Furthermore, in immune cells, treatment of C3a has been found to mediate adhesion of eosinophils to cytokine-primed endothelial and epithelial cells via integrin mediated interactions (96, 97). Adhesion of leukocytes to endothelial cells is an important step leading to diapedesis during cell migration. Apart from cell adhesion, C3aR activation has also been found to mediate chemotaxis of eosinophils and mast cells (98, 99). In some immune cells, C3aR-mediated chemotaxis is regulated via the Gαi signaling axis (100).

Although C3aR-mediated chemotaxis has been studied in several types of cells, little is known about its effects on chemotaxis and signal transduction in human macrophages. To date, C3aR-mediated chemotaxis has only been studied in the mouse macrophage cell line J774 (101). Various *in vivo* mouse studies suggest that C3aR activation plays an important role in inducing mouse macrophage infiltration and activation to key metabolic organs such as adipose tissues and kidneys (74, 102, 103). The analysis of C3aR-mediated chemotaxis and inflammation in human monocytes and macrophages is of increasing significance as previous studies have demonstrated the involvement of both C3aR expression and macrophage infiltration in various disease pathologies including arthritis, bacterial meningitis, atherosclerosis, and diabetes (74, 102–106). C3aR-mediated inflammatory responses have only been studied in human monocytes (107) and C3aR activation has shown to increase ATP efflux, NLRP3 inflammasome activation and IL1β secretion in human monocytes (107). With the new discoveries of potent and selective human C3aR agonists and antagonists, further studies on C3aR function in human macrophages will provide important insights (108).

### Complement Protein 5a Receptor (C5aR)

Cleavage of complement protein C5 produces complement peptide C5a (70). Similar to C3a, complement peptide C5a is a potent proinflammatory, and chemotactic factor that also acts via an inflammatory GPCR to trigger immune cell activation (71). The C5a receptor C5aR was recently renamed C5aR1 and is expressed widely on immune cells, including monocytes, macrophages, eosinophils, neutrophils and T cells. Additionally C5aR is expressed on non-hemopoietic cells in the liver, kidney, adipose tissue, the central nervous system, and other tissues (109). C5aR inhibition blocks inflammation in various mouse models or can reverse established pathology in the K/B × N mouse arthritis model (72). Thus, therapeutic interventions that target C5aR are currently investigated in diseases of inflammatory etiology (110) as well as cancer (111).

Only a few potent and selective low molecular weight peptidomimetic and small molecule C5aR antagonists have been reported (73). These antagonists have shown anti-inflammatory activity *in vitro* in human immune cells including macrophages and efficacy *in vivo* in animal models of inflammatory disease (73). C5aR antagonists 3D53, W54011, and JJ47 all show potent inhibition of C5a-induced chemotaxis and inflammatory gene expression in human and rat macrophages (73). Further C5aR has shown to differentially modulate LPS-induced inflammatory responses in human monocytes and macrophages (112). While C5aR activation potentiated LPS-induced inflammatory mediators in human monocytes, C5a inhibited these responses in human M1-like and M2-like macrophages (112). Interestingly, C5aR activation also enhanced clearance of the Gram-negative bacterial pathogen Salmonella enterica serovar Typhimurium from human macrophages (112). However, despite the importance of C5aR in macrophage biology and the success in preclinical models of human inflammatory diseases, few C5aR antagonists have so far progressed beyond phase I clinical trials.

### Protease-Activated Receptors

Protease activated receptors, PARs (PAR1-4), are an unusual group of cell surface GPCRs, which self-activate after cleavage of their N-termini by pre-dominantly danger-associated serine proteases to enable coupling and activation of intracellular G-protein signaling cascades (113). Among PARs, PAR2 is the most highly expressed PAR on immune cells like human macrophages and is activated by multiple proteases involved in inflammation (74). Various extracellular pathogen-associated (e.g., house dust mite and cockroach proteases, Gingipains, and others) and damage-associated (thrombin, trypsin, tryptase, tissue factor VIIa, kallikreins, neutrophil elastase, cathepsin G, proteinase 3, and others), serine proteases are sensed by PAR2 to trigger intracellular signaling and inflammation (74). In human macrophages, PAR2 gene expression and cell-surface protein expression are increased by LPS and common dietary fatty acids such as palmitic, stearic, and myristic acids (74). PAR2 activation is generally proinflammatory both *in vitro* and *in vivo*.

*In vitro*, PAR2 activation amplified palmitic acid-induced IL-1β and IL-6 secretion from human M2-like macrophages and a potent PAR2 antagonist GB88 prevented the augmented effect of IL-1β and IL-6 secretion by PAR2 activation (74). Furthermore, in both M1-like and M2-like human macrophages, the PAR2 activation increased TNFα and IL10 secretion in a manner similar to LPS (114). Interestingly, human monocytes that were matured to an M1 phenotype in the presence of a PAR2 agonist had a reduced cell area, and released less TNF-α after LPS challenge (75). In contrast, human monocytes matured to an M2 phenotype in the presence of a PAR2 agonist also had a reduced cell area and made significantly more TNFα after LPS stimulation (75). Furthermore, a recent study suggests that PAR2 activation promotes M1 macrophage polarization and inflammation via activating the FOXO1 pathway in mouse macrophages (115). *In vivo*, the PAR2-activating protease, tissue factor VIIa, acting via PAR2 activates adipose tissue macrophages, and causes insulin resistance and metabolic dysfunction in mice (76). In another independent study, the PAR2 antagonist, GB88, given orally to diet-induced obese rats also completely attenuated adipose tissue inflammation and prevented increased infiltrated macrophages into the adipose tissue (116). Taken together, these *in vitro* and *in vivo* studies suggest that among PARs, PAR2 might play an important role in macrophage differentiation and activation. However, no PAR2-directed therapies have yet been devised for treating various macrophage-driven chronic inflammatory disease conditions.

## Other Macrophage-Expressed GPCRs (Table 2)

### Histamine H_4_ Receptor (HRH4)

HRH4 is a Class A histamine receptor that was initially cloned and characterized as a result of a databank search and found to be localized in blood and peripheral lymphoid organs (123). The receptor was shown to be expressed specifically by natural killer cells and cells of the myeloid linage (124). In macrophages, it is exclusively upregulated after *in vitro* stimulation of cells with GM-CSF while monocytes and CSF-1 differentiated macrophages are virtually negative for this receptor (7). This observation that GM-CSF is required for HRH4 expression was supported by the finding that dendritic cells expressed this receptor after differentiation from monocytes using this cytokine in combination with IL-4 (125). The role of histamine in inflammatory conditions such as allergies and the expression on cells of the immune system have generated a strong interest in HRH4 as a drug target. Recent studies have demonstrated a strong anti-inflammatory function of HRH4 inhibitors in psoriasis and lung inflammation (117).

**Table 2:** Other macrophage-expressed GPCR.

**Full name**	**Receptor** **abbreviation**	**Class**	**Function (macrophage)**	**References**
Histamine H_4_ receptor	HRH4	Class A	Potentially macrophage activation (M2) and immune regulation	(117)
5-Hydroxytryptamine receptor 2B or serotonin receptor 2B	HTR2B	Class A	Potentially macrophage polarization (M2)	(118)
EGF-like module-containing mucin-like hormone receptor-like (EGF-TM7 family)	EMR3	Class B	Potentially macrophage activation and immune regulation	(119)
Angiotensin receptors	AT1 – AT4	Class A	Pro- and anti-flammatory macrophage activation	(120, 121)
Succinate receptor 1	SUCNR1	Class A	M2 polarization, metabolic response of obesity	(122)
Mas-related GPCR	MRGPCRF	Class A	Potentially inflammation	(7)
Endothelin receptor	ETA, B1, B2, C	Class A	Potentially macrophage polarization and inflammation	(7)
Lysophosphatidic acid receptor	LPA_5_ or GPR92	Class A	Potentially macrophage activation and inflammation	(7)
G protein-coupled receptor family C type 5A	GPCR5A	Class C	Inflammation	(7)

### 5-Hydroxytryptamine Receptor 2B (5-HT_2B_) and 7 (5-HT_7_)

HTR are a receptor family for the neurotransmitter 5-Hydroxytryptamine (serotonin) that belongs to the Class A family of GPCRs (10). Two members of this family 5-HT2B and 5-HT7 have been identified to be expressed strongly on M2 macrophages with both receptors skewing the differentiation to the M2 activation state (118). In an independent study it could be shown that 5-HT2B while weakly expressed on monocytes and GM-CSF macrophages was strongly upregulated by CSF-1 stimulation (7). While a large number of small molecule agonists and antagonists for these receptor family have been established and have been tested in clinical trials, none have been identified that target macrophages specifically.

### EGF-Like Module-Containing Mucin-Like Hormone Receptor-Like 3

EMR3 belongs to the EGF-TM7 family of receptors. All family members are class B adhesion GPCRs (10) and expressed exclusively on cells of the immune system (126). In humans EMR3 is present on myeloid cells including CD16^+^ monocytes (127) and strongly upregulated after GM-CSF differentiation (7). While the expression of adhesion GPCRs has been demonstrated on macrophages in atherosclerotic plaques and autoimmune inflammatory pathologies such as MS no clear understanding of their function has been published yet (119).

### Angiotensin Receptor 1 and 2

The two extensively studied angiotensin receptors AT1 and AT2 and two less characterized receptors (AT3 and AT4) are class A GPCRs that are activated by the octapeptide effector hormone Angiotensin II (Ang2). They are best known for their role in regulating blood pressure and are targeted clinically in the treatment of hypertension (128). AT1 and AT2 are expressed on activated monocytes/macrophages and Ang2 has been shown to provide a powerful proinflammatory stimulus via AT1 (120). In contrast, the specific activation of AT2 on LPS-activated human monocytic cell lines provides an anti-inflammatory signal and causes a downregulation of the expression of proinflammatory cytokines such as TNF (121). Since macrophages and their expression of cytokines have been shown to play an important, yet not entirely understood role in hypertension these divergent roles of the angiotensin receptors could potentially be used to develop a highly specific therapy for this pathology (129).

### Succinate Receptor 1

In a recent publication, compelling evidence has been presented that the Succinate receptor 1 (SUCNR1) is involved in M2 polarization of adipose fat-associated macrophages. Thus, this GPCR is part of the regulatory system that controls the metabolic response to obesity (122).

### Other GPCRs

The Class A receptors Mas-related GPCRs (MRGPCRsF), endothelin receptor (EDNRB), Lysophosphatidic acid receptor 5 (LPA_5_ or GPR92) and G protein-coupled receptor family C type 5A (GPCRs5A) are strongly expressed after macrophage differentiation (7) but have not yet been studied in detail in the context of macrophage function.

## Roles of GPCRs in Tissue-Resident Macrophages

A multitude of GPCRs with ligands active in macrophage development and localization (chemokine receptors) and inflammation (biogenic amines (Histamin), lipid mediators (prostaglandins, prostanoids, leukotrienes, platelet-activating factor), peptide hormones (Chemokines, C5a, anaphylatoxin) have been described to be expressed on mouse and human macrophages (6, 7, 61). The sheer number of GPCRs expressed on macrophages and the large variety of tissue-resident macrophage populations make a comprehensive review not feasible. Therefore, we will focus on cardiac and tumor-associated macrophages as examples for emerging therapeutic targets.

### Cardiac Macrophages

Cardiovascular disease is despite intensive research and new treatment options the leading reason for mortality worldwide. The role of macrophages in the maintenance of cardiovascular health has not been fully appreciated and only recently the contribution of the innate immune system has begun to become better understood (130–133). In the healthy heart macrophages of embryonic origin are maintained by local proliferation (134). They contribute to tissue homeostasis and regeneration by phagocytosis of dead cells and tissue repair, produce locally a variety of factors such as cytokines and have been shown to form connections to cardiomyocytes which allows them to support electrical conduction within cardiac tissue (131, 132). This population co-exists with monocyte derived CCR2^+^ macrophages and fate analysis has demonstrated that these monocyte-derived macrophages replace successively with age macrophages of embryonic origin (135).

After an ischemic myocardial injury a large number of monocytes egresses the spleen and infiltrates the cardiac tissue (136). The release from the spleen reservoir is not CCR2-dependent as demonstrated in CCR2-deficient mice but monocyte accumulation in the myocardium is lacking in this model (136). An interesting role has been shown for the angiotensin receptor 1 (AGTR1) using Atgr1a^−/−^ animals (136) Atgr1a is present on activated monocytes (137, 138) and in gene-deficient mice the egress from the spleen is rendered ineffective and reduced significantly (136). Since the influx of monocytes has to be carefully timed to be beneficial and to contribute to tissue healing after infarction both receptors, CCR2 and AGTR1 could be a therapeutic target.

### Tumor Associated Macrophages (TAMs)

Solid tumors that have become detectable clinically have evaded immune surveillance. This is astonishing given that up to 3% of the tumor mass consists of TAMs. The underlying mechanisms how the immune response is subverted are still not understood entirely (49) but a study of renal cell carcinoma has shown a negative correlation of TAMs and regulatory T cells with long term survival (139). It a meta-analysis of various other cancers such as breast, bladder, ovarian, oral and thyroid cancer it has been confirmed that the density of TAMs in tumor tissue allows a negative prognosis of the overall survival (140). In contrast, a positive effect could be detected in patients with colorectal cancer (140). In a small pilot study no significantly different effect of M1 vs. M2 macrophages could be detected. Therefore, the role of macrophage polarization in human TAMs remains controversial (140).

These observations that demonstrate that the presence of macrophages has a negative effect on the outcome of cancer are consistent with earlier studies that showed that the chemokine CCL2 which is expressed by tumor cells and stroma drives the recruitment. TAMs express vascular endothelial growth factor A in the tumor environment and facilitate extravasation of cells from the tumor thus promoting metastasis (141). A blocking of the CCL2/CCR2 axis using an anti-CCL2 agent in a mouse model reduces the number of infiltrating monocytes by preventing them from leaving the bone marrow and inhibits metastasis significantly (141). This points a potential therapeutic approach that could be used to prevent the spreading of metastatic tumor cells to secondary tissues. An unexpected side effect of this therapeutic approach became apparent when the treatment of CCL2 was interrupted (142). This caused an enhanced mobilization of cells from tumors and an increased the number of metastases (142). Nevertheless, antibody-based biologicals and small molecule inhibitors specific for CCR2 are currently used in trials for cancer treatment and other anti-inflammatory therapies (10). Instead of blocking TAM infiltration an alternative approach is desirable that aims at specific macrophage functions or activities that support tumor growth. An example would be the presence of large quantities of extracellular adenosine in tumors. These nucleotides have been demonstrated to modify macrophage activation (143). Activation of macrophages in the presence of adenosine which acts through the A2A receptor results in the M2 phenotype causing in immunosuppression (144). Use of inhibitors of purinergic receptors in combination with checkpoint therapy could be a useful new approach to cancer therapy (145).

## Conclusion

The complexity of the possible functional interactions of these monocyte/macrophage receptors with the micro- and macro-environment is elevated by various factors. First, macrophages are a highly heterogeneous family of cells that are able to respond to changes in the tissue environment with remarkable plasticity (146). While reductionistic *in vitro* approaches have been able to form a picture of specific gene signatures of macrophage populations after stimulation with opposing cytokines such as GM-CSF and CSF-1 or IL-4 and IFN-γ (32, 36) it is highly unlikely that under inflammatory conditions *in vivo* equivalent populations can develop in adjacent tissue areas depending on the respective conditions. In fact, recent, unbiased single cell, deep sequencing experiments of microglia demonstrated an impressive heterogeneity in the unchallenged brain and were able to distinguish six different microglia populations (95). One can speculate that in inflammation a similar approach would show a vast and confusing number of macrophage populations displaying different signatures due to concurrently ongoing activation, differentiation and polarization.

Second, GPCRs e.g., LGR4 which are expressed on macrophages show an overlapping expression in various tissues such as skin and kidneys (84) while the expression of other receptors e.g., EMR3 or CCR2 is limited to cells of the hemopoietic linage. Receptors with an expression in the myeloid line such as F4/80 (EGF-TM7 family) or CCR2 have shown a specific non-overlapping function knockout studies in mouse models (147, 148). How this translates to the function of macrophages in a transient blocking in humans *in vivo* using small molecule antagonists has not yet been experimentally evaluated. Developing macrophage specific gene deficient mouse models could be a first step to a better understanding of off-target effects of an interference with specific receptors in complex models such as infection.

Finally, other complicating factors are tissue specific gene regulation of GPCRs using different promoter structures (149) and the possibility of an alternative splicing of these receptors for example in the EGF-TM7 family of receptors resulting in various final proteins (150). Overall, the broad range of these receptors and the complexity of expression on macrophage subsets may present challenges in effectively targeting them in order to modulate macrophage activity for the treatment of disorders involving these phagocytes. However, given that there have been successes resulting in licensing of some agents, this should be encouraging for further development and applications. It is interesting to note that other small molecule targeted therapies have shown unexpected but potentially useful off-target effects. One example being abl kinase inhibitors for chronic myeloid leukemia, such as Imatinib, having the ability of inhibiting c-fms, the M-CSF/CSF-1 receptor, with inhibitory effects on monocyte development, macrophage, and osteoclast activity (151, 152). However, further work will be necessary to unveil the full potential of a therapeutic targeting of GPCRs on macrophages.

## Author Contributions

WW contributed to determine the content of each section and edited the manuscript. XW, AI, AL, and HK collected data and wrote the each section of the manuscript. All authors read and approved the final manuscript.

### Conflict of Interest Statement

The authors declare that the research was conducted in the absence of any commercial or financial relationships that could be construed as a potential conflict of interest.
